# Commentary: Tensiomyographic Markers Are Not Sensitive for Monitoring Muscle Fatigue in Elite Youth Athletes: A Pilot Study

**DOI:** 10.3389/fphys.2017.01068

**Published:** 2017-12-13

**Authors:** Saúl Martín-Rodríguez, Damir Zubac, Francisco Piqueras-Sanchiz, Iker J. Bautista, Bostjan Simunic

**Affiliations:** ^1^Colegio Oficial de Licenciados en Educación Física de Canarias, Las Palmas, Spain; ^2^Science and Research Centre Koper, Institute for Kinesiology Research, Koper, Slovenia; ^3^Head of Sport Lab, Sport Plus Center, Sevilla, Spain; ^4^FisioSalud Elite, Health, Training & Innovation, University of Granada, Granada, Spain

**Keywords:** muscle contractile properties, fatigue, training monitoring, junior athletes, high intensity interval training

With great interest, we read the article by Wiewelhove et al. (2017). This article contributes to the on-going debate regarding the usefulness of tensiomyography (TMG) in the detection of peripheral muscle fatigue (Wiewelhove et al., 2015; de Paula Simola et al., 2016; Macgregor et al., 2016). In this article, the main conclusion was that TMG was not sensitive to detect significant muscular performance changes and consequently, any muscle fatigue induced by a high intensity interval training (HIT) protocol. Although the authors used appropriate statistics' descriptors to analyze the diagnostic accuracy of the TMG measures for the assessment of muscle fatigue, we suggest that some issues within the manuscript need to be considered before adopting these conclusions and such a resounding title. In this regard, we acknowledge the transparency of the authors when reporting the study's limitations, but we feel that such limitations drastically compromised their results.

We acknowledge that the article is a “*pilot study*,” however, it is insufficient to obtain the conclusions raised by the authors at a methodological level. There are some flaws in the calculations made in Table 2 of the article regarding the effect size (ES) and *p*-value of the “V_10_” variable (ES = −0.32, *p* = 0.225) since we have recalculated it and obtained different results [ES = −0.68; −1.18; −0.17, 95% confidence interval (CI)]. We suggest that the authors review the inferential statistics performed since the CI of this variable does not cross the zero line. We argue that if a larger sample size was tested, then significant changes in TMG parameters [maximal displacement (Dm); rate of deformation development until 10% (V_10_) and 90% (V_90_) Dm, respectively] would have been observed following the HIT microcycle (as evidenced by our Table 1 calculations) and consequently muscle fatigue would have been detected. Since *post-hoc* power analysis is no longer recommended (Hoenig and Heisey, 2001), 95% CI were calculated (not included by the authors) and are presented in Table 1 since CI replace calculated power (ß) once a study is done (Wilkinson, 1999). In addition, a Bayesian approach to interpret statistical significance was added (Table 1) using a web platform (http://www.graphpad.com/) to observe if Type II error occurred. Power was calculated using G^*^Power software (version 3.1) taking their current TMG results. The 95% CI and Bayesian calculations of Dm, V_10_ and V_90_ indicate that the chance of association was >75% if the sample size was larger. The above confirms Type II error and indicates that Wiewelhove and colleagues should have performed *ad hoc* power analysis to be confident about their results. Finally, it seems that contraction time (Tc) would not have changed as previously observed (Wiewelhove et al., 2015).

**Table 1:** 95% CI and Bayesian calculation and interpretation of author's results at 80% prior probability.

**TMG parameter**	**M ± SD (95% CI) pre-post differences**	***p-*value**	**ß**	**Bayesian calculation**		**Bayesian interpretation**	
				**Posterior Odds**	**Posterior probability**	**% chance no association**	**% chance association**
Dm	−0.7 ± 0.1 (−0.76; 0.64)	0.178	0.36	8.09	0.89	24.31	75.69
Tc	0.2 ± 0.7 (0.2; 0.60)	0.896	0.05	0.22	0.18	2.66	97.34
V_10_	−2.8 ± 3.3 (−4.7; −0.89)	0.225	0.45	8.00	0.89	16.29	83.71
V_90_	−12.4 ± 5.3 (−15.46; −9.34)	0.189	0.38	8.04	0.89	24.64	75.36

The author's results showed no significant alterations in the TMG parameters following completion of the 4-day HIT program, however, a significant decline in CMJ height was observed. Although CMJ is considered a valid and reliable method to assess an athlete's fatigue (Markovic et al., 2004), this test is not able to assess localized muscle fatigue. Furthermore, CMJ performance may not be limited by RF muscle fatigue, since Wong et al. (2016) recently showed that RF plays a marginal role during the push-off phase of vertical jumping and other muscles (vastii, gastrocnemii, hamstrings) are much more important for maximizing jump height. Therefore, the measures that Wiewelhove et al. implemented to assess peripheral muscle fatigue (lower limb muscle soreness and CMJ height) likely reflect significant fatigue of other muscles, but not the RF. This is not surprising as many other hip/knee extensor and flexor muscles are involved in sprint-related exercises (Morin et al., 2015). If these other muscles were tested with TMG we argue that TMG markers would have been sensitive enough to detect peripheral muscle fatigue. This is because we have found that the TMG response of various lower limb muscles is different following training microcycles, competitions and bed-rest (Pisot et al., 2008; García-Manso et al., 2011; Rodríguez-Matoso et al., 2015). Collectively, drawing conclusions on mechanisms of neuromuscular fatigue built around single muscle assessment should be considered controversial and the arguments about TMG's effectiveness put forward by Wiewelhove et al. are misleading because it is unlikely that significant muscle fatigue was present in the RF following their HIT protocol with the current sample size.

In conclusion, if the authors had recruited a larger sample size, it is likely that TMG would have been effective at detecting RF muscle fatigue, as evidenced by our Table 1 calculations. A greater sample size was likely necessary because there was only some RF fatigue following the HIT protocol. However, it is also true that the test employed to detect fatigue (i.e., CMJ) of the lower limb muscle analyzed (i.e., RF) was not appropriate according to Wong et al. (2016). Thus, we encourage the authors to carry out an investigation that includes better tests to detect fatigue and compare with TMG responses from multiple muscles.

## Author contributions

SM-R conceived, drafted, and revised the manuscript; DZ, FP-S, IB, and BS drafted and revised the manuscript. All authors read and approved the final manuscript.

### Conflict of interest statement

The authors declare that the research was conducted in the absence of any commercial or financial relationships that could be construed as a potential conflict of interest.
